# *Mycobacterium ulcerans* Persistence at a Village Water Source of Buruli Ulcer Patients

**DOI:** 10.1371/journal.pntd.0002756

**Published:** 2014-03-27

**Authors:** Martin W. Bratschi, Marie-Thérèse Ruf, Arianna Andreoli, Jacques C. Minyem, Sarah Kerber, Fidèle G. Wantong, James Pritchard, Victoria Chakwera, Christian Beuret, Matthias Wittwer, Djeunga Noumen, Nadia Schürch, Alphonse Um Book, Gerd Pluschke

**Affiliations:** 1 Swiss Tropical and Public Health Institute, Basel, Switzerland; 2 University of Basel, Basel, Switzerland; 3 FAIRMED Africa Regional Office, Yaoundé, Cameroon; 4 Bankim District Hospital, Bankim, Cameroon; 5 Labor Spiez, Spiez, Switzerland; Fondation Raoul Follereau, France

## Abstract

Buruli ulcer (BU), a neglected tropical disease of the skin and subcutaneous tissue, is caused by *Mycobacterium ulcerans* and is the third most common mycobacterial disease after tuberculosis and leprosy. While there is a strong association of the occurrence of the disease with stagnant or slow flowing water bodies, the exact mode of transmission of BU is not clear. *M. ulcerans* has emerged from the environmental fish pathogen *M. marinum* by acquisition of a virulence plasmid encoding the enzymes required for the production of the cytotoxic macrolide toxin mycolactone, which is a key factor in the pathogenesis of BU. Comparative genomic studies have further shown extensive pseudogene formation and downsizing of the *M. ulcerans* genome, indicative for an adaptation to a more stable ecological niche. This has raised the question whether this pathogen is still present in water-associated environmental reservoirs. Here we show persistence of *M. ulcerans* specific DNA sequences over a period of more than two years at a water contact location of BU patients in an endemic village of Cameroon. At defined positions in a shallow water hole used by the villagers for washing and bathing, detritus remained consistently positive for *M. ulcerans* DNA. The observed mean real-time PCR Ct difference of 1.45 between the insertion sequences IS2606 and IS2404 indicated that lineage 3 *M. ulcerans*, which cause human disease, persisted in this environment after successful treatment of all local patients. Underwater decaying organic matter may therefore represent a reservoir of *M. ulcerans* for direct infection of skin lesions or vector-associated transmission.

## Author Summary

Buruli ulcer (BU) is a neglected tropical disease caused by *Mycobacterium ulcerans* which affects mainly children in West Africa. Although it is commonly believed that the infection originates from an environmental source, both the reservoir of *M. ulcerans* and the mode of transmission to human patients remain to be elucidated. Previous investigations indicated that transmission likely takes place away from the homes of patients. We therefore screened the farms as well as village and farm water locations of 46 laboratory confirmed BU patients of the Mapé Basin of Cameroon for the presence of *M. ulcerans* DNA by real-time PCR. In this analysis three positive village water locations were identified. By studying one of these locations in great detail we found that *M. ulcerans* DNA persists in underwater detritus in one section of the village water location even after all local cases had been treated. The detritus may represent a reservoir of *M. ulcerans* from where infection could take place through either direct contamination of skin lesions or through contamination or colonization of insect vectors.

## Introduction

Buruli ulcer (BU) is a neglected tropical disease of the skin and subcutaneous tissue caused by the environmental pathogen *Mycobacterium ulcerans*. The disease, which can affect all age groups and both sexes, has been reported in over 30 countries but is most frequent in West Africa. Typically, BU presents with ulcers with undermined edges but clinical manifestations also include nodules, oedema and plaques. Lesions can encompass entire limbs if patients report late for treatment [1]. The WHO recommends that all cases should be laboratory confirmed by microscopy, polymerase chain reaction (PCR), primary culturing or histology [2]. However, because of the limited access to laboratory facilities in BU endemic areas, cases are often diagnosed based only on clinical signs and there is a pressing need for a simple, sensitive and specific point-of-care diagnostic test [3]. Historically, BU was treated using wide scale excision of the lesions. Since 2004, the WHO recommends a combination therapy of daily streptomycin and rifampicin for 8 weeks as the standard treatment for BU [1].

In Africa, the major risk factor for BU is proximity to stagnant or slow flowing water, but other factors such as poor wound care, and failure to wear protective clothing have also been identified in case-control studies [4]. It has further been reported, that man-made modifications of the environment may increase the incidence of BU [4]. Despite relentless efforts, both the reservoir and the exact mode of transmission of BU remain a mystery. Numerous investigations of the environment have attempted to identify the source of the pathogen with so far only limited success. Studies in Ghana and Benin, have examined environmental samples for the presence of the *M. ulcerans* insertion sequence (IS) 2404. Some of these studies have identified many IS2404 positive sites and found positive samples in both BU endemic and non-endemic areas [5]. On the other hand, a study from Ghana has reported that only very few samples were real-time PCR positive [6]. These difficulties to conclusively identify the environmental reservoir of *M. ulcerans* and the fact that investigations on its genome have revealed that the pathogen has undergone substantial niche adaptation [7], [8], have led investigators to look for invertebrate or vertebrate animal reservoirs [4], [7]. Specifically the role of aquatic insects as potential reservoirs has been evaluated [9], [10] and a recent study analyzing transmission networks has found that a specific taxa of aquatic invertebrates may be involved in the transmission of BU [11]. While to date no mammalian reservoir has been detected in Africa, possums have been identified as an animal reservoir of *M. ulcerans* in the southern Australian BU endemic area [12]. The mode of transmission from an animal or environmental reservoir to human patients also remains to be elucidated. Both insect bites, from mosquitos or water bugs, and direct inoculation of bacteria into the skin from an environmental reservoir after skin trauma have been suspected to be relevant for transmission [4] and several parallel modes of transmission may need to be considered [13].

The objective of the current study was to longitudinally monitor environmental contact water sources of laboratory confirmed BU patients for the persistence of *M. ulcerans* DNA.

## Materials and Methods

### Ethical statement

Approval for this study was obtained from the Cameroon National Ethics Committee (N°041/CNE/DNM/09 and N°172/CNE/SE/2011) and the Ethics Committee of Basel (EKBB, reference no. 53/11). Participation was voluntary and all patients, independent of their study participation, were treated according to national treatment guidelines. All cases who participated in the study or their legal guardian provided written informed consent.

### Study area, patient inclusion and patient confirmation

All real-time PCR confirmed cases identified in the Mapé Basin of Cameroon [13] between the beginning of December 2009 and the end of November 2011, were eligible for inclusion in this study. For definitive BU diagnosis, clinical samples were collected, DNA extracted and IS2404 real-time PCR performed as previously described [13]–[15]. Environmental sampling was performed between February 2011 and June 2013.

The main water bodies of the study area are the Mapé Dam and the Mbam River [13]. The region experiences two rainy seasons, a short one from mid-March to mid-May and a long one from mid-June to the end of September, with the rest of the year being dry.

### Selection of environmental sampling locations and sampling procedures

Patients selected for in-depth investigation were interviewed to determine where they lived for the year before the onset of BU. Homes of as many non-participating real-time PCR confirmed cases as possible were also identified and mapped. If participating patients had a home both in their village and at their farm, an interview was used to determine where they spent more time. After achieving an accuracy of less than 10 m, a GPS receiver was used to map the patient's home. Together with the patient, a close friend or relative, locations of regular environmental contact of the patient were then visited. The investigated and mapped locations included the patient's farm(s) and the location(s) where she/he obtained water while at home (VW: village water sources) or at the farm(s) (FW: farm water sources). Locations used to obtain water for drinking, cooking, bathing, clothes washing and dish washing were visited. At all locations, soil and plant material was collected. At the water contact locations, a water sample was also collected. Samples collected at the farms were dry soil and plants growing on dry grounds. Plant and soil samples from the water contact locations, were collected from either in the water, at the water's edge or in the moist area around the water.

Additionally, animal fecal samples were collected in the highly BU endemic village of Mbandji 2. Samples were collected around the homes of laboratory confirmed BU patients and included the feces of chickens, ducks, pigs, goats and sheep.

At two water contact locations located in Mbandji 2 (VW12 and VW13) we performed repeated and in-depth sampling over a period of more than two years (Table S1). In addition to the samples collected at the initial time point (t = 0) as described above, samples were collected from VW12 and VW13 at seven additional time points (t = 2.1, 4.8, 7.7, 10.5, 15.3, 20.3 and 27.4 months). At the two initial time points, samples were collected from 3 sampling sites at each VW location. At the remaining time points, samples were collected at 21–22 sampling sites around VW12, with 3–5 sample replicates at each sampling site. At the same time points, VW13 was sampled at 14–16 sampling sites with 1–3 sample replicates collected at each sampling site. Details of the sampling sites and the number of replicates collected at each sampling site and at each time point are given in Table S1. All samples, with the exception of those collected at sampling sites 7 and 13, which were plants on dry soil, were collected from inside the water or at the water's edge.

At the last follow-up time point (t = 27.4 months), further soil samples from inside the water were collected around the log at location VW12. At each sampling site 3–5 replicates of the same type of sample were collected. At several sampling sites on either side of the log, samples were repeatedly collected in the course of a few days. At sampling site 55, additional samples of various natures were collected.

All environmental samples were stored at 4°C until analysis.

### Environmental DNA extraction and real-time PCR

From the environmental samples, DNA was extracted and real-time PCR performed as previously described [14], [15]. Briefly, approximately 200 µL of each soil, plant and fecal sample was transferred to a lysing tube and DNA extracted using the Fast DNA Spin Kit for Soil (MP Biomedicals, product number 116560-200) and a Precellys24 homogenizer (Bervet Bertin Technologies). From the plant samples, DNA was extracted from a mixture of leaves and stems and if possible roots. For water samples, 1 mL was transferred to a lysing tube, the tubes centrifuged for 10 min (14'000 rpm), the supernatant removed and the samples then processed like the other samples. All samples were at least once extracted by the above method. Some samples were also processed once by homogenizing them in lysing matrix E tubes in the presence of MT Buffer (MP Biomedicals) and Phosphate Buffered Saline (MP Biomedicals), pelleting debris (10 min at 14'000 rpm) and then extracting DNA from the supernatant with the QIASymphony (Qiagen) and the QIAsymphony DSP Virus/Pathogen Midi Kit (Qiagen, product number 937055). The two different extraction methods yielded comparable DNA quantities, as assessed by real-time PCR, when applied in parallel to positive environmental samples (data not shown). For each extraction, a reagent control was included.

Extracted DNA (1 µL of 100 µL) was run twice in the IS2404 real-time PCR assays as previously described [14], [15]. In the IS2404 real-time PCR, an internal positive control (IPC, Applied Biosystems) was included to detect PCR inhibition. Inhibited samples were diluted 1/5 and 1/10 and analyzed again. In each real-time PCR run both negative and positive controls were included. If a sample was positive in at least one of the IS2404 real-time PCR assays, DNA was extracted from a second aliquot of the same environmental sample. If again at least one of two parallel IS2404 real-time PCR assays was positive, the corresponding environmental sample was considered positive for IS2404. DNA extracted from these samples (1 µL and 5 µL) was then subjected to IS2606 and keto reductase (KR) real-time PCR as previously described [14], [15]. If the extracts of a particular sample, were at least once positive for these two additional targets, the sample was considered positive for *M. ulcerans* DNA. All IS2404 positive samples that were not positive for both of the other targets were not considered to contain *M. ulcerans* DNA and were not included in the analysis. Most of the real-time PCR assays were performed in a StepOne Plus Real-Time PCR System (Applied Biosystems) and analyzed using the StepOne Software (v2.2.2; Applied Biosystems). Only samples extracted using the QIASymphony as well as samples analyzed in Cameroon, were real-time PCR tested for the presence of IS2404 by a Mastercycler Realplex 4 ep Gardient S (Eppendorf) and the data analyzed by Mastercycler ep Realplex (version 2.2).

### Statistical data analysis

Descriptive statistics were computed using R (The R Foundation for Statistical Computing; version 2.15.1) and RStudio (RStudio, Boston, USA; version 0.95.262). Maps were drawn in ArcGIS ArcMap (Economic and Social Research Institute, Redlands, USA; version 10.0).

## Results

### Screening of environmental contact locations of BU patients for the presence of *M. ulcerans* DNA

**Figure 1:**
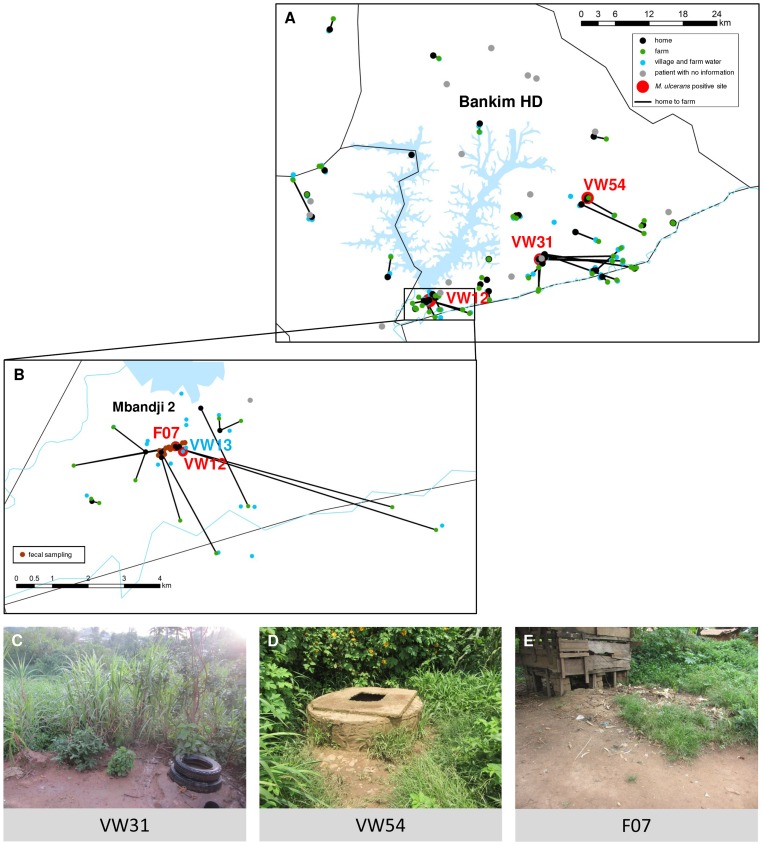
Environmental contact network of laboratory confirmed BU patients from the southern Mapé Basin. Panel A and B (detailed view of the village of Mbandji 2) show the houses where the 46 laboratory confirmed BU patients in our study lived (black points), the farm(s) where they worked (green points) and the locations where they obtained their water (blue points) during the year before the onset of BU symptoms. The home of each patient is connected with their farm(s) as applicable. Homes of 17 of the 21 non-participating laboratory confirmed BU patients were also mapped and are shown in grey. At the farms and water contact locations, soil (n = 171), plant (n = 153) and water (n = 109) samples were collected. Furthermore, in Mbandji 2 (B), animal faecal samples were collected around patients' houses (brown points). All samples were tested for the presence of *M. ulcerans* DNA and three village water locations were found to be positive (red points; VW12, VW31 and VW54). Further, at location F07 a positive duck faecal sample (red point) was collected. Photographs of locations VW31, VW54 and F07 are shown in C, D and E, respectively. Finally, Panel B also shows a negative water contact location (VW13) which was studied in detail.

From December 2009 to November 2011, 67 real-time PCR confirmed cases of BU were identified in the Mapé Basin of Cameroon. Of these patients, 46 were selected for in-depth environmental contact analysis based on their origin in the southern part of the Mapé Basin and their availability to participate in the study. The homes and farms as well as the VW and FW locations of the patients were mapped (Figures 1A and 1B). The median direct distance between the homes and farms was 1.5 km (interquartile range = 0.6 km to 5.3 km). While some patients lived permanently at their farm, others travelled more than 15 km to get from their home to their farm (Table 1). As shown in Figures 1A and 1B, many of the BU patients in the southern Mapé Basin moved south and east towards the Mbam River for their farming activities.

**Table 1:** Environmental contact locations of laboratory confirmed BU patients tested for *M. ulcerans* DNA.

Patient ID	Village Water (VW) ***	Farm (F) ***	Farm Water (FW) ***	Distance Home – F (km)
**01**	VW01, VW02, VW03	F not visited	FW01, FW02	NA
**02**	VW04, VW05	F01	NA *	0.63
**03**	VW06, VW07	F02	FW03	12.14
**04**	VW08, VW09	F03	FW04, FW05	14.31
**05**	VW10, VW11	F04	FW06	5.62
**06**	VW12#, VW13	F05, F06	FW07	7.54; 6.19
**07**	VW14, VW15, VW16	F07, F08	NA *	0.34; 0.65
**08**	VW17	F09	FW08	0.69
**09**	VW18, VW19	F10	FW09, FW10	3.03
**10**	VW20, VW21	F11	FW11	2.27
**11**	VW22	F12	FW12	1.08
**12**	VW23, VW24	F13, F14, F15	FW13	5.37; 5.26; 1.40
**13**	VW12#, VW13	F16	FW not visited	2.87
**14**	VW25	F17	FW14	1.57
**15**	VW26	F18	FW15, FW16	3.20
**16**	VW27, VW28	F19	FW17, FW18	11.96
**17**	VW29	F20	FW19, FW20	3.51
**18**	VW30	F21	FW21	0.55
**19**	VW31#	F22	FW22	12.62
**20**	VW32	F23	NA *	0
**21**	VW not visited	F24	FW23	0
**22**	VW33	F25, F26	FW24	0.89;1.20
**23**	VW34, VW35	F27	FW25	2.00
**24**	VW36, VW37	F not visited	FW not visited	NA
**25**	VW38	F28	FW not visited	5.66
**26**	VW39	F29	FW26, FW27	1.48
**27**	VW40, VW41	F30	FW28, FW29	7.32
**28**	VW42, VW43	F31	FW30, FW31, FW32, FW33	4.40
**29**	VW44, VW45, VW46	F32 **	FW34	3.60
**30**	VW47	F33	FW35	15.35
**31**	VW48	F34	NA *	0.30
**32**	VW49	F35	NA *	0.99
**33**	VW50	F36	FW36	1.83
**34**	VW12#, VW13	F16	FW not visited	2.98
**35**	VW51	F37, F38	NA *	0.08; 0.21
**36**	VW52, VW53	F39	NA *	0.38
**37**	VW54#	F40	NA *	0.24
**38**	VW55	F41	FW37	1.31
**39**	VW56, VW57	F42	NA *	0.59
**40**	VW58, VW59	F43, F44	FW38, FW39	1.13; 0.84
**41**	VW52, VW53	F45	NA *	0.79
**42**	VW60	F46	NA *	0
**43**	VW61	F47	NA *	0.02
**44**	VW not visited	F48	FW40, FW41, FW42	5.91
**45**	VW62	F49	FW43	13.52
**46**	VW48	F50	NA *	0.17

NA: not applicable.

* Water carried to farm from home.

** Location not tested by real-time PCR.

*** VW, F and FW locations are individually numbered; locations which are shared between patients are identified by the same number.

# Positive for *M. ulcerans* DNA.

Environmental samples (171 soil, 153 plant and 109 water samples) were collected at the farms (n = 49), FW (n = 43) and VW (n = 48) locations shown in Figure 1A. Of the soil and plant samples, 108/171 and 109/153 respectively, were collected in or around water. The remaining samples were collected from dry grounds. All environmental contact locations are numbered in Table 1; locations used by several patients are indicated by the same number. Additionally, pig, goat, sheep, chicken and duck fecal samples (n = 24) were collected at 14 sampling sites in the BU endemic village of Mbandji 2 (Figure 1B).

All environmental and fecal samples were tested by real-time PCR for the presence of the *M. ulcerans* specific IS2404 DNA sequence. Three VW locations (VW12, VW31 and VW54) and one duck fecal sample (F07) tested positive (Figures 1A and 1B). At locations VW31 (Figure 1C) and VW54 (Figure 1D), soil samples collected in the moist area around the water wells were positive. Both of these water locations were used by one BU patient each (Table 1). Water from VW31 was reported to be used for bathing and washing of clothing and water from VW54 was used for all purposes including drinking. At location VW12, used by three of the patients living in the village of Mbandji 2, both a soil and a plant sample collected at the water's edge, tested positive. Further details on the results of a longitudinal study at VW12 are provided below.

### Persisting real-time PCR positivity of detritus after successful treatment of the identified local BU patients

**Figure 2:**
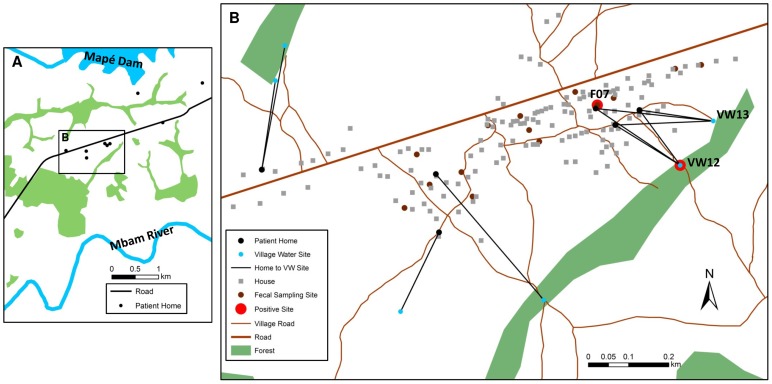
Water contact locations in Mbandji 2 which were investigated in detail. Based on the high case number and the identification of two environmental locations which were positive for *M. ulcerans* DNA, water contact locations in Mbandji 2 were analysed in detail. The town is located between the Mapé Dam and the Mbam River (A). Panel B shows the locations of the homes of the 6 patients from Mbandji 2 in our study (black points) and each of the homes is connected with the village water location(s) used by the respective patient. Faecal sampling sites are also shown (brown points). Locations which tested positive for *M. ulcerans* DNA are highlighted in red (B). A positive (VW12) as well as a close by negative (VW13) village water locations were studied in more detail. Images are based on a 0.5 m resolution WorldView-2 image take on March 12th 2011.

As shown in Figures 1B and 2A, six BU patients were notified during the study period in Mbandji 2, which is situated between the Mapé Dam and the Mbam River (Figure 1E). The locations of the homes of these patients are shown in Figure 2B and characteristics of the patients, which are not related to each other, are listed in Table 2. Patients 06, 13, and 34, aged 9, 5 and 57, respectively, all used the real-time PCR positive VW12 location (Figure 2B and Table 1). Furthermore, the only positive faecal sample (F07; from a duck) was collected in close proximity of the home of patient 13 (Figure 2B). The other three patients from Mbandji 2 used primarily four other VW locations (Table 2, Figure 2B).

**Table 2:** Laboratory confirmed BU patients in Mbandji 2 during the course of the study.

Patient ID	Age	Gender	Clinical Form	Category	Disease Start Date*	Discovery Date	Treatment Start	VW used by the patient
**06**	9	M	nodule	1	unknown	13.04.2010	24.07.2010	VW12 and VW13
**13**	5	F	plaque	2	11.09.2010	06.11.2010	10.11.2010	VW12 and VW13
**15**	2	M	ulcer	3	26.10.2010	30.11.2010	03.12.2010	VW 26
**33**	11	M	ulcer	2	10.03.2011	05.05.2011	10.05.2011	VW 50
**34**	57	M	ulcer	1	01.03.2011	10.05.2011	12.05.2011	VW12 and VW13
**40**	42	M	ulcer	3	06.09.2009	07.08.2011	07.08.2011	VW 58 and 59

* Calculated based on information provided by the patient.

**Figure 3:**
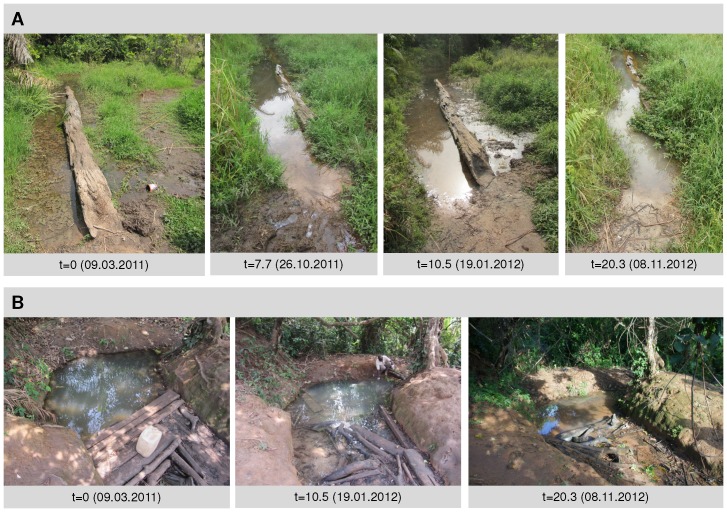
Alterations of the environment at locations VW12 and WV13 at the sampling time points. Photographs of locations VW12 (A) and VW13 (B) are shown at selected environmental sampling time points.

To better characterize IS2404 real-time PCR positivity in Mbandji 2, we performed detailed longitudinal analyses of VW12 and the close-by IS2404 negative location VW13 (Figure 1B and 2B). VW12 was a permanent small water body with a wooden log lying in it (Figure 3A). The water was shallow and flowed slowly from the left to the right when approaching the log from the village of Mbandji 2. For most of the log, the left and right side of the water were not connected under the log; however at some points water could pass underneath the log. On the right side of the log, the vegetation was denser and a layer of detritus was accumulating. In contrast, the compacted ground on the left was not covered with detritus. Location VW12 was used by the local population – including patients 06, 13 and 34 (Table 1 and 2) – to wash clothing and for bathing. For these activities, locals stood in the water on the left side of the log. The father of patient 13 also reported that his daughter went to this location to play. In contrast, VW13 was used by the local population – including again patients 06, 13 and 34 – to obtain drinking water as well as water for cooking and bathing. In the front section of VW13, where there were planks of wood (Figure 3B), water emerged from several springs.

**Figure 4:**
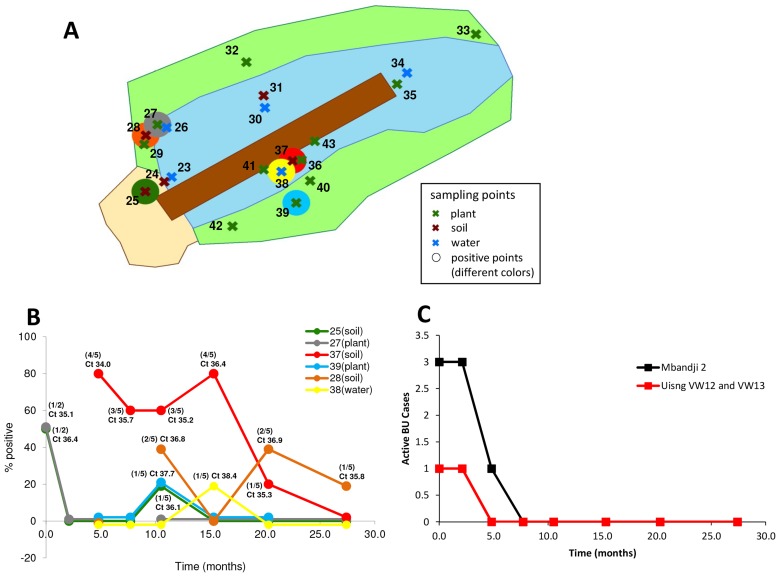
Persistence of *M. ulcerans* at a village water location of BU patients. Panel A shows a diagram of the water hole at VW12A from where samples were collected at eight time points over a period of 27.4 months. Soil sampling sites are shown as brown crosses, water sampling sites as blue crosses and plant sampling sites as green crosses. Table S1 shows how many samples were collected at each sampling site and each time point. All samples were tested for the presence of *M. ulcerans* DNA by real-time PCR. At 7 sampling time points, *M. ulcerans* real-time PCR positive samples were identified at VW12 (B with positive sampling sites identified by the larger coloured circles and C). Panel C (line colours correspond to the circle colours in panel B) shows the rate of positivity of the collected sample replicates as well as the average Ct value for the IS2404 real-time PCR performed on the positive samples. Finally, panel C shows the number of active BU cases in the village of Mbandji 2 (black line) and the number of active BU cases using VW12 (red line) at the environmental sampling time points.

We collected and analysed environmental samples at eight time points over a period of 27.4 months at both VW12 (n = 635) and VW13 (n = 217) (Table S1). Particularly at location VW12, substantial seasonal alterations of the environment over the study period were observed (Figure 3A and 3B). None of the 217 samples collected at VW13 tested positive in the IS2404 real-time PCR and only one of 108 samples taken from the sand pits, which are located immediately to the north-west of VW12 and are part of the larger VW12 location, tested positive (Figure 4B and 4C, Figure S1 and Table S1). In contrast, at 7/8 time points, positive samples were obtained from at least one of the six positive sampling sites identified at VW12 (Fig. 4). In particular underwater detritus samples collected at sampling site 37 were positive at 5/6 time points tested (Figures 4A and 4B). The average IS2404 real-time PCR Ct values of the positive samples varied between 34.0 and 38.4 (Figure 4B). As shown in Figure 4C, at the initial sampling time point, there was still one active case of BU (patient 34) using VW12 and there were still a total of three active BU cases in the entire village of Mbandji 2. However, from the third sampling time point on, no active BU case was using VW12 and from the fourth time point on, no active BU case was present in the entire village of Mbandji 2 (Figure 4C). Taken together environmental IS2404 real-time PCR positivity at the VW12 location thus persisted for more than one year after successful treatment of all BU patients identified in the village of Mbandji 2 (Figure 4).

**Figure 5:**
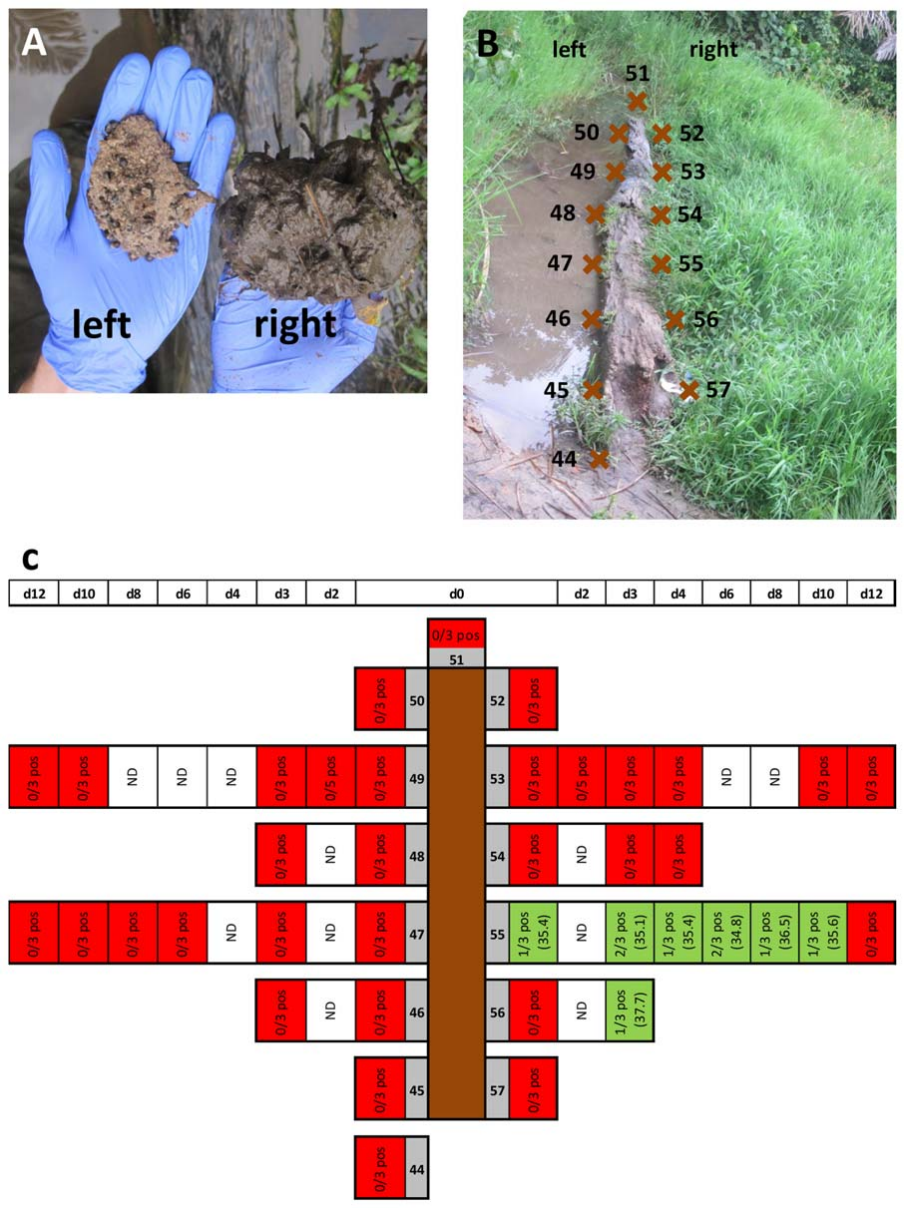
In-depth analysis of soil surrounding the log at VW12. Panel A shows the nature of the soil on the right and the left hand side of the log. To better understand positivity of samples at location VW12, we performed sampling all along the log on either side (B; brown crosses indicate sampling sites). Selected sampling sites were re-sampled over the next 12 days as indicated in panel C. Panel C further shows the rate of positivity among the replicates collected at each sampling time point and the average Ct value of the IS2404 real-time PCR performed.

Having identified the deposit on the right hand side of the log lying in VW12 as an IS2404 hotspot (Figure 4C), we analysed the soil all around the log in more detail. While compacted and sandy ground was found on the left, the ground was covered with decaying organic matter on the right hand side of the log (Figure 5A). At the eighth sampling time point we collected three replicates of soil samples every 1.14 m at a total of 14 sampling sites all around the log (Figure 5B). Using on site real-time PCR, we identified sampling site 55 (Figure 5C) as being positive (data not shown) and then sampled this location as well as other sampling sites around the log repeatedly over the next 12 days (Figure 5C). While all 59 samples collected on the left hand side and at the back of the log were negative, 9/62 samples from the right side of the log were positive. Positive samples were identified at sampling site 55 and the neighbouring sampling site 56 (Figure 5C) with an average IS2404 real-time PCR Ct value of 35.8 for all nine positive samples (Figure 5C) Additional sample types, including plants, roots or samples from the surface of the log, collected at sampling site 55 all tested negative (data not shown).

All the above mentioned IS2404 positive samples also tested positive in the IS2606 and the KR real-time PCR and the mean Ct difference (ΔCt) between IS2606 and IS2404 (IS2606-IS2404) of the samples was 1.45 (95% confidence interval from 1.10 to 1.80). This ΔCt indicated that the obtained PCR signal is not related to lineage 1 *M. ulcerans*, which are fish and frog pathogens, or lineage 2 *M. ulcerans*, both of which harbor only few copies of IS2606, but that the PCR signal we observed likely originates from lineage 3 *M. ulcerans*, which are found in human lesions and contain 63–98 copies of IS2606 per genome [7].

## Discussion

In African BU endemic areas, both the nature of the environmental reservoirs of *M. ulcerans* and the mode of transmission to humans have so far remained unclear. The physical environment, e.g. biofilms, and organisms such as amoeba, insects, fish and frogs have all been proposed as possible reservoirs for the pathogen [4]. Investigations in Southern Australia have identified mammals, specifically possums, as a local reservoir of *M. ulcerans* [12]. However, no such mammalian source of the pathogen has been detected in Africa thus far [16]. As for the transmission to humans, hypotheses include insect vectors and direct inoculation from the environment via small skin lesions. Parallel modes of transmission may, depending on the environmental and epidemiological setting, be relevant [4], [13]. A recent review on BU transmission, found that more evidence is needed to conclude that insects are involved in *M. ulcerans* transmission [4]. Interestingly, *M. marinum*, the closest relative and ancestor of *M. ulcerans*, occasionally causes human infection by inoculation through small skin lesions which are often not remembered by the patient because of the long incubation period [17].

Although BU may occur at all ages, the relative risk for children below the age of five to develop the disease is lower than for older children [13], [18]. This appears to apply across different endemic areas in Africa and may indicate that exposure to *M. ulcerans* is increasing, once children are taking up new activities away from their homes [13], [19]. Such activities could include going to the farm to work or to water sources for household activities, to collect water or to play. With this in mind and since proximity to water bodies undoubtedly is a risk factor for BU [4], we set out to systematically test environmental, and in particular water, contact locations of laboratory confirmed BU patients. Specifically we collected plant, soil and water samples at the farms as well as village and farm water locations of patients and tested them for the presence of *M. ulcerans* DNA.

Due to the abundance of other and faster growing microorganisms in the environment, routine cultivation of *M. ulcerans* from environmental samples has mostly failed [20] and to date only a single *M. ulcerans* isolate from a water-strider, has been reported [21]. Attempts to culture from our samples were not successful also because of the overgrowth by other mycobacteria. By PCR using pan-mycobacterial and hsp65 primers [22], [23] and DNA sequencing, we detected species such as *M. shimoidei*, *M. psychrotolerans* and *M. chubuense* in our preparations (data not shown). Because of these difficulties, real-time PCR for IS2404 is commonly used to detect *M. ulcerans* in the environment. We applied most stringent quality control procedures with internal positive controls in each sample as well as negative and positive controls in each real-time PCR run. Further, we only considered an environmental sample positive if it was positive in two separate DNA extractions. With this approach we are confident that the positive samples truly contain *M. ulcerans* DNA. We can however not exclude, particularly given the heterogeneity of the environmental samples, that some positive samples may be missed. Although IS2404 is considered a specific marker for *M. ulcerans* [24], the existence of IS2404 positive *M. ulcerans* ecotypes (lineage 1) that are largely avirulent for humans complicates interpretation of real-time PCR data and requires that samples are also tested for the presence of IS2606 and that the difference between the IS2606 and the IS2404 Ct value is analyzed [7], [14]. Because *M. ulcerans* ecotypes that cause human disease in Africa and Australia (lineage 3) harbor a higher number of IS2606 sequences then those of linage 1, the ecovars can be separated based on the IS2606 to IS2404 ΔCT [7]. All 41 IS2404 positive environmental samples collected in the course of this study also tested positive for IS2606 with a mean IS2606 to IS2404 ΔCt of 1.45. This ΔCt is well below the ΔCt of 7 to 8 of the for humans less virulent lineage 1 *M. ulcerans* strains [14]. Two IS2404 and IS2606 positive samples tested negative in the real-time PCR for the lower copy number virulence plasmid associated KR sequence. Both of these samples, which had relatively high Ct-values for the IS2404 and IS2606 real-time PCR, were not included in the list of *M. ulcerans* DNA positive samples discussed in this paper.

Our screening of environmental contact locations of laboratory confirmed BU patients revealed that they travel considerable distances to get to their farms and some of the patients further reported to spend several months there. Molecular typing studies of disease isolates may help to identify if the patients were infected close to their homes or farms [25].

By testing environmental samples, we identified two *M. ulcerans* DNA positive water wells (VW31 and VW54) in two different villages. In a third village we identified an *M. ulcerans* positive duck fecal sample (F07) and a positive open permanent water location (VW12). While this rate of environmental positivity is similar to what has been found in a study from Ghana [6], positivity was much higher in another study also conducted in Ghana [26]. It is interesting to note that all three positive locations were permanent as opposed to seasonal water sources. Obtaining water from such water sources has previously been shown to increase the risk for BU [27]. The positive duck fecal sample, merits further investigation to determine how waterfowl may contribute to the reservoir of *M. ulcerans*.

At VW31 and VW54 we did not investigate the local scenario any further and cannot exclude the possibility that these locations were contaminated with *M. ulcerans* DNA from the lesions of patients living close to the wells. However at VW12, a water source used by laboratory confirmed BU patients from Mbandji 2, we observed longitudinal persistence of *M. ulcerans* DNA in underwater detritus for more than one year after successful treatment of the last BU patient. Continuous presence and case search in the village allowed us to detect all local cases and it is therefore unlikely that the source of the environmental positivity was from bacteria recently spread from a human lesion. Interestingly, the more sandy ground on the left of the log at WV12 was never real-time PCR positive for *M. ulcerans* DNA and even on the right side of the log the distribution of *M. ulcerans* DNA was highly focalized (Figures 4 and 5), with samples taken from sampling sites just a few meters apart giving different results. The persistent of real-time PCR positivity in the detritus is a strong indication that this micro-environment may represent a niche environment to which *M. ulcerans* has adapted in the course of evolution from the more generalist *M. marinum* [7], [28]. How these findings are related to the recently identified potential role of aquatic worms in BU transmission [11] should be investigated further.

The previously described age distribution of BU patients in the Mapé Basin [13] and the here described findings of *M. ulcerans* DNA at village water sources, lead to the hypothesis that around the age of four both exposure to *M. ulcerans* and the risk of contracting BU increases. At this age children are beginning to be sent to fetch water and may get in direct contact with the environmental source of the pathogen. Our data further suggest that, underwater detritus could represent a reservoir of *M. ulcerans*, from where infection could take place through either direct contamination of skin lesions or through contamination or colonization of insect vectors.

## Supporting Information

Figure S1 **Sampling sites at VW13 and the sand pits at VW12.** Diagram of VW13 and the sand pits close to VW12 with the sampling sites; soil sampling sites are shown as brown crosses, water sampling sites as blue crosses and plant sampling sites as green crosses. For details on the main water body of VW12 (transparent part) see Figure 4.
(TIF)

Table S1 **Number of environmental samples collected at each sampling sites of VW12 and VW13 at all sampling time points.** Environmental samples (soil, plant and water) were collected at eight time points over a period of 27.4 months at up to 43 sampling sites at the two locations VW12 and VW13. The table shows how many sample replicates were collected at each sampling site and each time point.
(DOCX)
