# Supplementary figures and images for: Mycobacterium ulcerans Persistence at a Village Water Source of Buruli Ulcer Patients
Source: PLoS Negl Trop Dis. 2014 Mar 27;8(3):e2756. doi: 10.1371/journal.pntd.0002756 (PMC3967953; doi:10.1371/journal.pntd.0002756)

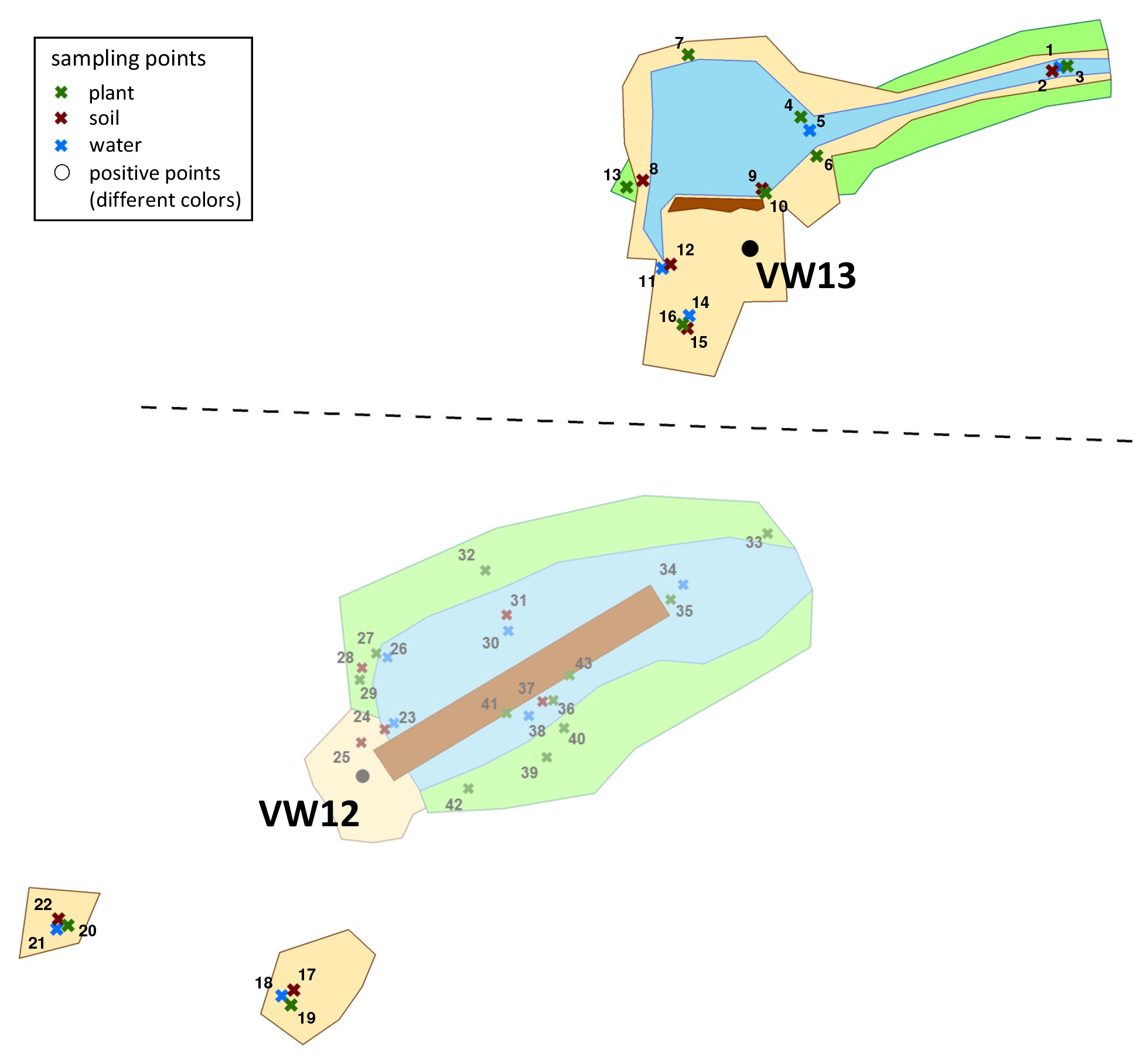

Supplement: Figure S1 — Sampling sites at VW13 and the sand pits at VW12. Diagram of VW13 and the sand pits close to VW12 with the sampling sites; soil sampling sites are shown as brown crosses, water sampling sites as blue crosses and plant sampling sites as green crosses. For details on the main water body of VW12 (transparent part) see Figure 4. (TIF) [file pntd.0002756.s001.tif]
