# Supplementary material for: Mycobacterium ulcerans Persistence at a Village Water Source of Buruli Ulcer Patients
Source: PLoS Negl Trop Dis. 2014 Mar 27;8(3):e2756. doi: 10.1371/journal.pntd.0002756 (PMC3967953; doi:10.1371/journal.pntd.0002756)
Supplement: Table S1 — Number of environmental samples collected at each sampling sites of VW12 and VW13 at all sampling time points. Environmental samples (soil, plant and water) were collected at eight time points over a period of 27.4 months at up to 43 sampling sites at the two locations VW12 and VW13. The table shows how many sample replicates were collected at each sampling site and each time point. (DOCX) [file pntd.0002756.s002.docx]

**Table S1: Number of environmental samples collected at each sampling sites of VW12 and VW13 at all sampling time points.**

| **Sampling Site*** | **Sample Type** | **t = 0** | **t = 2.1 months** | **t = 4.8 months** | **t = 7.7 months** | **t = 10.5 months** | **t = 15.3 months** | **t = 20.3 months** | **t = 27.4 months** |
| --- | --- | --- | --- | --- | --- | --- | --- | --- | --- |
| **1** | water | NA | NA | 1 | 3 | 3 | 3 | 3 | 3 |
| **2** | soil | NA | NA | 1 | 3 | 3 | 3 | 3 | 3 |
| **3** | plant | NA | NA | 1 | 3 | 3 | 2 | 3 | 3 |
| **4** | plant | NA | NA | 1 | 3 | 3 | 3 | 3 | 3 |
| **5** | water | 1 | NA | 1 | 3 | 3 | 3 | 3 | 2 |
| **6** | plant | 1 | NA | 1 | 3 | 3 | 3 | 3 | 3 |
| **7** | plant | NA | NA | 1 | 3 | 3 | 3 | 3 | 3 |
| **8** | soil | NA | NA | 1 | 3 | 3 | 3 | 3 | 3 |
| **9** | soil | NA | NA | NA | NA | 3 | 3 | 3 | 3 |
| **10** | plant | NA | NA | 1 | 3 | NA | NA | NA | NA |
| **11** | water | NA | NA | 1 | 3 | 3 | 3 | 3 | 3 |
| **12** | soil | NA | NA | 1 | 3 | 3 | 3 | 3 | 3 |
| **13** | plant | NA | NA | 1 | 3 | 3 | 3 | 3 | 3 |
| **14** | water | NA | NA | 1 | 3 | NA | NA | 3 | 3 |
| **15** | soil | 1 | NA | NA | 3 | NA | NA | NA | NA |
| **16** | plant | NA | NA | NA | NA | 3 | 2 | 3 | 3 |
| **17** | soil | NA | NA | 5 | 3 | 3 | 3 | 3 | 3 |
| **18** | water | NA | NA | 4 | 3 | NA | 3 | 3 | 3 |
| **19** | plant | NA | NA | 5 | 3 | 3 | 3 | 3 | 3 |
| **20** | plant | NA | NA | 5 | 3 | 3 | 3 | 3 | 3 |
| **21** | water | NA | NA | 4 | 3 | 3 | 3 | NA | 2 |
| **22** | soil | NA | NA | 5 | NA | 3 | 3 | 3 | 3 |
| **23** | water | 1 | 1 | NA | 5 | NA | NA | 5 | NA |
| **24** | soil | NA | NA | 5 | NA | 5 | 5 | NA | 5 |
| **25** | soil | 2 | 1 | 4 | 5 | 5 | 5 | 5 | 5 |
| **26** | water | NA | NA | NA | 5 | NA | NA | NA | NA |
| **27** | plant | 2 | 3 | 5 | NA | 5 | 5 | 5 | 5 |
| **28** | soil | NA | NA | 5 | NA | 5 | 5 | 5 | 5 |
| **29** | plant | NA | NA | NA | 5 | NA | NA | NA | NA |
| **30** | water | NA | NA | 5 | 5 | 5 | 5 | 5 | 3 |
| **31** | soil | NA | NA | 5 | 5 | 5 | 5 | 5 | 5 |
| **32** | plant | NA | NA | 5 | 5 | 5 | 5 | 5 | 5 |
| **33** | plant | NA | NA | 5 | 5 | 5 | 5 | 4 | 5 |
| **34** | water | NA | NA | 4 | 5 | 5 | 5 | 5 | 5 |
| **35** | plant | NA | NA | 5 | 5 | 5 | 4 | 5 | 5 |
| **36** | plant | NA | NA | 5 | 4 | 5 | 5 | 5 | NA |
| **37** | soil | NA | NA | 5 | 5 | 5 | 5 | 5 | 5 |
| **38** | water | NA | NA | 5 | 5 | 5 | 5 | 5 | 5 |
| **39** | plant | NA | NA | 5 | 5 | 5 | 5 | 5 | NA |
| **40** | plant | NA | NA | 5 | 5 | 5 | 5 | 5 | 5 |
| **41** | plant | NA | NA | 5 | 5 | 5 | 5 | 5 | 5 |
| **42** | plant | NA | NA | 5 | 5 | 5 | 5 | NA | 5 |
| **43** | plant | NA | NA | 5 | 5 | 5 | 5 | 4 | 5 |

* Sampling sites 1 to 16 are at VW13 and 17 to 43 are at VW12.
